# The Antioxidant 3H-1,2-Dithiole-3-Thione Potentiates Advanced Glycation End-Product-Induced Oxidative Stress in SH-SY5Y Cells

**DOI:** 10.1155/2012/137607

**Published:** 2012-05-17

**Authors:** Robert Pazdro, John R. Burgess

**Affiliations:** ^1^Department of Nutrition Science, Purdue University, West Lafayette, IN 47907, USA; ^2^The Jackson Laboratory, 600 Main Street, Bar Harbor, ME 04609, USA

## Abstract

Oxidative stress is implicated as a major factor in the development of diabetes complications and is caused in part by advanced glycation end products (AGEs). AGEs ligate to the receptor for AGEs (RAGE), promoting protein kinase C (PKC)-dependent activation of nicotinamide adenine dinucleotide phosphate (NADPH) oxidase and superoxide radical generation. While scavenging antioxidants are protective against AGEs, it is unknown if induction of endogenous antioxidant defenses has the same effect. In this study, we confirmed that the compound 3H-1,2-dithiole-3-thione (D3T) increases reduced-state glutathione (GSH) concentrations and NADPH:quinone oxidoreductase 1 (NQO1) activity in SH-SY5Y cells and provides protection against H_2_O_2_. Surprisingly, D3T potentiated oxidative damage caused by AGEs. In comparison to vehicle controls, D3T caused greater AGE-induced cytotoxicity and depletion of intracellular GSH levels while offering no protection against neurite degeneration or protein carbonylation. D3T potentiated AGE-induced reactive oxygen species (ROS) formation, an effect abrogated by inhibitors of PKC and NADPH oxidase. This study suggests that chemical induction of endogenous antioxidant defenses requires further examination in models of diabetes.

## 1. Introduction

Oxidative stress is a primary component of diabetes pathology [1] and is considered a causal factor for the development of complications like neuropathy [2], which is characterized by hyperalgesia and sensory dysfunction [3–5]. Antioxidants suppress oxidative stress and ameliorate symptoms of diabetic neuropathy in experimental systems [6, 7], and while antioxidants have the potential to provide clinical benefit [8], more studies are required to properly investigate the effectiveness of antioxidants in humans.

 Concentrations of reactive oxygen species (ROS) are elevated in diabetes due to increased mitochondrial superoxide radical generation and depletion of endogenous antioxidant systems [9–11]. ROS are also the result of advanced glycation end products (AGEs), which are produced by the nonenzymatic glycation of proteins [12, 13]. Carboxymethyl lysine (CML)—a regularly formed product of protein glycation [14]—ligates to the receptor for AGEs (RAGE) [15], facilitating activation of extracellular signal-regulated kinase (ERK) [16], p38 kinase [17], and the transcription factor NF-kappaB [17]. In neuronal cells, RAGE ligation increases protein kinase C (PKC)-dependent nicotinamide adenine dinucleotide phosphate (NADPH) oxidase activity and superoxide radical generation [18, 19]. This pathway of ROS generation causes apoptosis and macromolecule damage *in vitro* [19, 20] as well as functional neurological deficits *in vivo* [21–23].

 Antioxidants such as probucol [23], *α*-tocopherol [24], *α*-lipoic acid [25], and N-acetyl-cysteine (NAC) [23, 25, 26] provide cytoprotection against AGEs. NAC exerts its protective effects by increasing intracellular GSH [27, 28]; studying alternative methods of increasing intracellular GSH might highlight additional mechanisms that protect neurons from AGE-induced damage. One such mechanism is through activation of the transcription factor Nrf2, which is responsible for constitutive and inducible upregulation of antioxidant enzymes, including those responsible for GSH regulation. Nrf2 activity can be stimulated by chemical compounds from the diet, including dithiolethiones, which are sulfur-containing compounds found in cruciferous vegetables. One of the dithiolethiones, 3H-1,2-dithiole-3-thione (D3T), potently upregulates antioxidant genes in cells and *in vivo* [28, 29] by activating Nrf2 [30]. Because D3T-mediated protection involves increased GSH biosynthesis [28], we hypothesized that D3T would confer protection against AGE-induced oxidative stress.

In this paper, we used SH-SY5Y cells to confirm previous reports that D3T increases intracellular GSH levels as well as activity of the antioxidant enzyme NADPH:quinone oxidoreductase 1 (NQO1). We report that D3T pretreatment partially suppressed H_2_O_2_-induced cytotoxicity. However, D3T potentiated harmful effects of AGEs. D3T pretreatment caused greater AGE-induced cytotoxicity and GSH depletion, and it had no effect on protein carbonylation and neurite degeneration. We found that D3T caused higher rates of AGE-induced ROS formation, an effect suppressed by inhibitors of PKC and NADPH oxidase. In all, D3T potentiates the toxic effects of AGEs in a cell culture model of diabetic neuropathy.

## 2. Materials and Methods

### 2.1. Materials

Cell culture supplies, including Dulbecco's modified Eagle's medium (DMEM), Ham's F12 media, fetal bovine serum (FBS), and penicillin-streptomycin, were obtained from Invitrogen (Carlsbad, CA, USA). Materials for AGE-BSA formation, including fatty acid-free BSA and glycolaldehyde, were obtained from Sigma-Aldrich (St. Louis, MO, USA). Retinoic acid (RA) and dimethyl sulfoxide (DMSO) were also purchased from Sigma-Aldrich. All cultureware was obtained from VWR (West Chester, PA, USA). SH-SY5Y cells were purchased from ATCC (Manassas, VA, USA). N-acetyl-cysteine (NAC) and *α*-tocopherol were obtained from Sigma-Aldrich. 3H-1,2-dithiole-3-thione (D3T) was purchased from LKT Laboratories (St. Paul, MN, USA).

### 2.2. Formation of AGE-BSA

AGE-BSA was prepared as described previously [31] with minor alterations. Briefly, BSA (5 mg/mL in PBS) was incubated with 33 mM glycolaldehyde dimer for 20 hours at 37°C. Glycated BSA was then dialyzed extensively for several days against PBS at 4°C. To concentrate the proteins to achieve high-dose treatments, AGE-BSA was precipitated using ammonium sulfate (Mallinckrodt Chemicals, Phillipsburg, NJ, USA). The protein precipitate was then reconstituted in a smaller volume of PBS and dialyzed again for several days against PBS at 4°C. Control (unglycated) BSA was treated the same except that the initial incubation was in PBS without glycolaldehyde. After the second dialyzing step, protein concentrations were quantified using a BCA protein assay kit (Pierce, Rockford, IL). Dilutions of 1 mg/mL were then measured for absorbance at 340 nm on a PowerWave plate reader (BioTek Instruments, Winooski, VT, USA). Fluorescence (335 nm excitation, 420 nm emission) was measured on a SpectraMax Gemini XS fluorescence plate reader (Molecular Devices, Sunnyvale, CA, USA). Control BSA and AGE-BSA stock solutions were aliquoted and frozen at −20°C.

### 2.3. Cell Culture and Treatment

SH-SY5Y cells were grown and maintained in 10% FBS and 1% penicillin-streptomycin in DMEM at 37°C and 7% CO_2_. For experiments, cells were counted using Trypan Blue (ATCC) staining and seeded in normal growth media. After allowing cells to adhere overnight, neurite outgrowth was encouraged with 10 *μ*M RA in DMEM : F12 (1 : 1) with 1% FBS for 3–5 days. For experiments using D3T, cells were pretreated with either D3T or DMSO vehicle control (0.1% DMSO) for 24 hours in DMEM : F12 (1 : 1) with 1% FBS and 10 *μ*M RA. After 24 hours, DMEM : F12 was removed and replaced with media containing 4 mg/mL BSA, AGE-BSA, or equivalent dilution of PBS. Treatments lasted for 16–24 hours, and cells were then harvested for specific experiments.

### 2.4. Cell Viability

Cell viability was assessed using the MTT assay [32]. Our preliminary work with this assay demonstrated that the changes in absorbance have high correlation with SH-SY5Y cell number as determined by trypan blue exclusion (*R* ^2^ = 0.996). A stock solution of 5 mg/mL 3-[4,5-dimethyl-2-thiazolyl]-2,5-diphenyl-2H-tetrazolium bromide (Sigma-Aldrich) in PBS was diluted in media to 0.5 mg/mL. After treatment, DMEM : F12 was removed and cells were incubated for 3 hours with MTT media. Purple formazan crystals that had formed in viable cells were dissolved by adding 20% SDS (Sigma-Aldrich) in 50% dimethylformamide (Sigma-Aldrich) followed by agitation on a plate shaker for one hour. Aliquots of 200 *μ*L were pipetted into a 96-well plate and read for a test absorbance of 570 nm and a background absorbance of 690 nm on a PowerWave plate reader (BioTek Instruments). Data were expressed as percentage of PBS control. In experiments involving D3T pretreatments, all values are expressed as percentage of PBS control with DMSO pretreatment.

### 2.5. GSH Measurement

Total intracellular GSH was determined after cell treatments. Cells were washed three times with PBS, scraped into ice-cold PBS, and centrifuged to form a cell pellet. The pellet was resuspended in 5% metaphosphoric acid + 100 *μ*M EDTA, and cells were lysed by several freeze-thaw cycles. A centrifugation step promoted the precipitation of proteins that were later quantified using a BCA protein assay kit (Pierce). The supernatant was filtered and analyzed by high-performance liquid chromatography (HPLC) with electrochemical detection. Specifically, the samples were injected into an ESA Coularray system (Chelmsford, MA, USA) with 5% mobile phase B (20% methanol, 30% mobile phase A, 50% acetonitrile) in mobile phase A (50 mM sodium phosphate, pH 3). The predominant peak for GSH had a potential of 900 mV and eluted at approximately 4.6 minutes. Samples were usually run the day in which they were prepared; if this was not possible, samples and standards were frozen at −80°C until they could be analyzed. GSH concentrations were normalized to total protein.

### 2.6. NQO1 Activity

NQO1 activity was measured following 24 hours of D3T pretreatment by using a previously published method [33] with some minor alterations. A quantity of 7.0 × 10^5^ cells was treated and washed twice with PBS and incubated in 500 *μ*L 0.8% digitonin, 2 mM EDTA, pH 7.8. The dishes were incubated for 10 minutes at 37°C and then shaken on an orbital shaker. Aliquots of 50 *μ*L were then used for analysis of NQO1 activity as indicated [33]. Similar to the MTT assay, NQO1 activity depends on the reduction of MTT to purple formazan crystals. NQO1 activity was calculated based on change in absorbance over a specified time period and with 11,300 M^−1^ cm^−1^ used as the extinction coefficient for reduced MTT at 610 nm. Additional aliquots were used for quantification of total protein by a Pierce BCA total protein kit.

### 2.7. Cell Morphology

SH-SY5Y cells were plated on collagen-coated cellware and treated with RA. Cells were then treated for 24 hours with either 0.1% DMSO or 50 *μ*M D3T. DMSO and D3T were removed and cells were treated with 4 mg/mL BSA, AGE-BSA, or an equivalent dilution with PBS for an additional 24 hours. Some AGE-BSA-treated samples also received 2.0 mM NAC. The morphologies of cells were documented using differential interference contrast (DIC) microscopy on an Olympus 1 × 70 inverted microscope. An Olympus DP70 digital camera system was used for image capture at 10x, 20x, and 40x. Images were obtained from at least four random fields in three independent samples per treatment. Digital images at 40x were saved as random numbers to allow for blinding, and neurites were counted using Image J software (NIH).

### 2.8. Protein Carbonyls

SH-SY5Y cells were pretreated for 24 hours with 0.1% DMSO or 50 *μ*M D3T and then treated for 24 hours with 4 mg/mL BSA, AGE-BSA, or an equivalent dilution with PBS. Cells were washed with ice-cold PBS three times and then scraped into cold RIPA buffer containing DTT. Centrifugation allowed for separation of insoluble protein components, and the concentration of soluble proteins in RIPA buffer was quantified using the Pierce BCA protein assay kit. Soluble proteins were derivatized using the OxyBlot protein oxidation detection kit (Chemicon, Temecula, CA, USA) according to the manufacturer's instructions to detect relative amounts of oxidized proteins. The derivatized proteins were separated on a 12.5% Tris-HCl precast gel (Bio-Rad Laboratories, Hercules, CA, USA). The oxidized proteins were detected by recommended antibody dilutions provided with the OxyBlot kit and with use of Amersham ECL advance western blotting detection kit (GE Healthcare Life Sciences, Buckinghamshire, UK). The intensities of protein bands were quantified by UN-SCAN-IT Gel 6.1 software (Silk Scientific, Orem, UT, USA). The data were standardized and expressed relative to PBS control with DMSO pretreatment.

### 2.9. Intracellular ROS Detection

 To measure intracellular H_2_O_2_ and hydroxyl radical concentrations, SH-SY5Y cells were cultured in a 24-well plate and pretreated for 16 hours with 0.1% DMSO or 50 *μ*M D3T followed by the addition of 2 *μ*M rottlerin or 20 nM DPI for the 30 minutes immediately prior to AGE-BSA or BSA treatment. BSA or AGE-BSA (4 mg/mL) was then added to each well. After 16 hours of incubation, 5(6)-carboxy-2′,7′-dichlorofluorescein diacetate (DCFH) (Sigma-Aldrich) was added to wells for a final concentration of 50 *μ*M. Cells were incubated for 30 minutes at 37°C and then washed twice with DMEM containing 1% FBS. Prepared media (400 *μ*L) was added to each well, and the fluorescence was read by a SpectraMax Gemini XS fluorescence plate reader with an excitation wavelength of 485 nm and an emission wavelength of 530 nm. A gain in fluorescence is indicative of DCFH oxidation [34]. The background fluorescence from empty wells was subtracted. Results were standardized to cell density, and data were expressed as a percent of PBS control with DMSO pretreatment.

### 2.10. Statistical Analysis

All experiments were repeated three times or more when specified. Statistical significance was determined by ANOVA. The Tukey test for multiple comparisons of groups was performed as post hoc analysis. Interactions with *P* values less than 0.05 were determined to be statistically significant.

## 3. Results

### 3.1. Influences of AGEs on Cell Viability

In this study, we confirmed previous findings that AGEs are cytotoxic to SH-SY5Y cells [35, 36]. AGE-BSA (4 mg/mL) decreased MTT absorbance approximately 35% in comparison to PBS controls (*P* < 0.0001, data not shown); unglycated BSA caused no negative effects. We also confirmed that the antioxidants NAC and *α*-tocopherol partially suppressed AGE-induced cell death (*P* < 0.05 and *P* < 0.0005 versus AGE-BSA, resp.; data not shown). Using higher concentrations of AGE-BSA than those in our previous studies did not alter our findings that AGE-BSA—and not control BSA—decreases cell viability, and antioxidants suppress this effect.

### 3.2. Effects of D3T on Antioxidant Defenses

We measured intracellular GSH concentrations and NQO1 activity to test the ability of D3T to upregulate endogenous antioxidant defenses. D3T pretreatments equal to or below 10 *μ*M did not affect GSH concentrations, but 25 *μ*M D3T increased intracellular GSH approximately 2.5-fold (*P* < 0.001 versus DMSO controls; data not shown) and 50 *μ*M D3T increased GSH approximately 3.5-fold (*P* < 0.0001 versus DMSO controls). Treatment with 25–50 *μ*M D3T also increased NQO1 activity. Because 25 and 50 *μ*M D3T increased measures of endogenous antioxidant defenses without negatively impacting cell viability (data not shown), we proceeded with 50 *μ*M D3T in follow-up studies. To confirm whether upregulation of endogenous antioxidant defenses by D3T increases stress resistance in our model, we pretreated SH-SY5Y cells with either 0.1% DMSO or 50 *μ*M D3T for 24 hours and followed this with 2 hours of incubation with 0, 200, or 500 *μ*M H_2_O_2_ (data not shown). In vehicle controls, 200 *μ*M H_2_O_2_ decreased viability approximately 40%, and 500 *μ*M H_2_O_2_ decreased viability approximately 65% (*P* < 0.0001 between 0, 200, and 500 *μ*M H_2_O_2_). In comparison, D3T increased cell viability approximately 20% in cells exposed to H_2_O_2_ (*P* < 0.0001 between D3T versus DMSO-pretreated cells at each H_2_O_2_ concentration). Therefore, upregulation of GSH and NQO1 activity by D3T protects SH-SY5Y cells against H_2_O_2_. We then examined the ability of D3T pretreatment to protect against the mitochondria complex I inhibitor rotenone. Rotenone caused an approximately 30% decrease in viability as assessed by MTT assay; D3T had no effect on rotenone-induced cell death (data not shown). In all, we confirmed previous findings by Jia et al. that D3T upregulates GSH and NQO1 in SH-SY5Y cells and this protection suppresses H_2_O_2_-induced cytotoxicity [37]. Although D3T upregulates endogenous antioxidant defenses in SH-SY5Y cells, our data suggest that the ability of D3T to promote oxidative stress resistance depends on the specific stressor.

### 3.3. Influence of D3T on AGE-Induced Cell Loss

**Figure 1:**
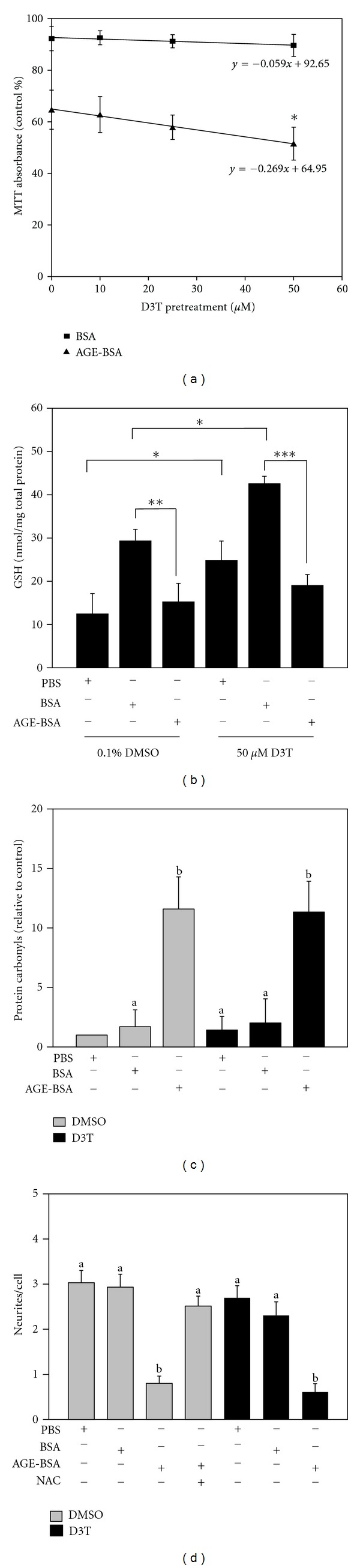
D3T potentiates AGE-induced cytotoxicity. (a) SH-SY5Y cells were pretreated for 24 hours with 0–50 *μ*M D3T followed by a 24 hr treatment with BSA or AGE-BSA. MTT was performed as indicated in the Materials and Methods section and absorbances were standardized to 0.1% DMSO treatment. Results are from three independent experiments (*n* = 8–14); bar values are expressed as mean ± SD. (b) GSH was measured by HPLC and standardized to total protein. Results are from four independent experiments (*n* = 4); bar values are expressed as mean ± SD (*P < 0.01; **P < 0.0005; ***P < 0.0001). (c) Cells were pretreated with 0.1% DMSO or 50 *μ*M D3T and then treated with 4.0 mg/mL BSA, AGE-BSA or an equivalent dilution of PBS. Protein oxidation was determined using an OxyBlot kit (Millipore). Band intensity was quanitified by UN-SCAN-IT Gel 6.1 software (Silk Scientific, Orem, UT, USA) and standardized relative to PBS control with DMSO pretreatment. Results are from three independent experiments; bar values are expressed as mean ± SD. (d) Cells were pretreated with 0.1% DMSO- or 50 *μ*M D3T and then treated with 4.0 mg/mL BSA, AGE-BSA ± 2.0 mM NAC, or an equivalent dilution of PBS. Neurite number was quantified using Image J software. Results are from three independent experiments (*n* = 3-4 random views); values are expressed as mean ± SEM. Data were analyzed by ANOVA. Superscript letters that are not the same are significantly different (*P* < 0.05).

 We pretreated SH-SY5Y cells with 0–50 *μ*M D3T and followed with 24 hours of incubation with 4 mg/mL BSA or AGE-BSA. Both 25 *μ*M and 50 *μ*M D3T increase endogenous antioxidant defenses, but here 25 *μ*M D3T-pretreatment caused an approximately 10% further decrease in viability after AGE-BSA treatment. However, this interaction only approached statistical significance (*P* < 0.10 versus 0.1% DMSO control + AGE-BSA). Surprisingly, 50 *μ*M D3T potentiated cytotoxicity caused by AGE-BSA (Figure 1(a); *P* < 0.0001 versus DMSO); yet the same concentration caused no negative impact on unglycated BSA-treated samples. Therefore, D3T potentiates the cytotoxic effects of AGE-BSA in a dose-dependent manner and with a mechanism that appears to be exclusive to this particular stress.

### 3.4. Effects of AGE-BSA and D3T on GSH Levels and Oxidative Damage

 We tested whether D3T affects AGE-induced oxidative damage and GSH depletion. We observed that BSA increased intracellular GSH compared to PBS controls. We also noted that D3T pretreatment increased GSH when compared to DMSO controls (*P* > 0.01; Figure 1(b)), except in AGE-BSA treated cells, where the difference between DMSO and D3T pretreatments was not statistically significant. Thus, in comparison to vehicle controls, D3T caused a larger AGE-induced decrease in GSH (*P* > 0.0001). In all, D3T increased intracellular GSH in controls, but this antioxidant potentiated the decline in GSH due to AGE-BSA, suggesting that D3T increases AGE-induced oxidative stress.

 In an effort to assess the effects of D3T on macromolecule oxidation, we examined protein carbonyl formation by Oxyblot kit. Neither PBS nor BSA caused substantial protein carbonylation in either DMSO or D3T-pretreated cells. AGE-BSA significantly increased protein carbonyl content (*P* > 0.0001 versus PBS and BSA controls). This trend was not affected by pretreatment with D3T (Figure 1(c)).

### 3.5. Influence of D3T on SH-SY5Y Cell Morphology

 NAC prevents AGE-induced neurite degeneration in SH-SY5Y cells [36], so we tested whether increasing GSH with D3T instead of NAC had the same protective effect. We confirmed that AGE-BSA (4 mg/mL for 24 hours) decreased the average number of neurites per cell while the BSA control had no effect (Figure 1(d)). Consistent with our previous work, NAC suppressed AGE-induced neurite degeneration. To assess the influence of D3T on these outcomes, we pretreated cells with 50 *μ*M D3T and then followed with 24 hours of treatment with 4 mg/mL BSA, AGE-BSA, or equivalent dilution with PBS. D3T had no independent effects on neurite number, but unlike NAC, D3T pretreatment did not protect the neurites of SH-SY5Y cells treated with AGE-BSA. While both NAC and D3T induced comparable GSH increases in SH-SY5Y cells, albeit through separate mechanisms, NAC maintained neurite number in cells exposed to AGE-BSA, and D3T provided no protection.

### 3.6. Influence of D3T on ROS Formation

**Figure 2:**
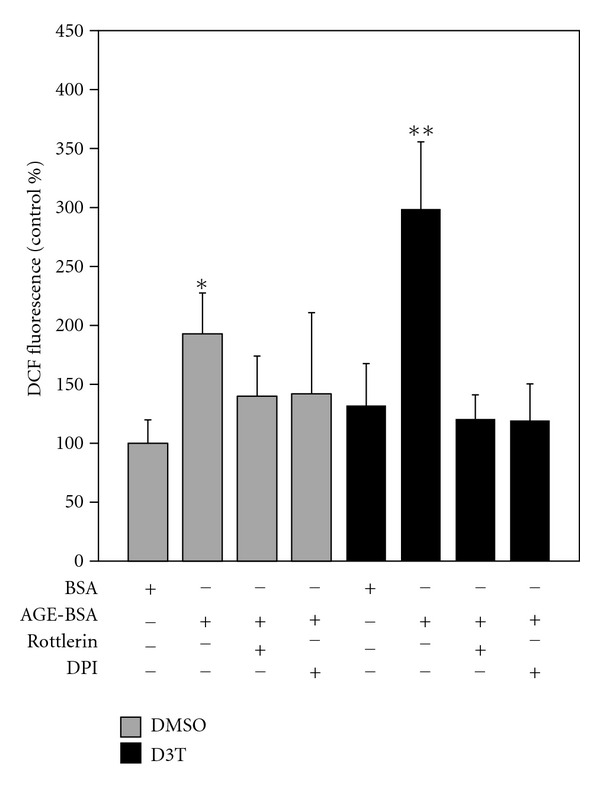
D3T potentiates AGE-induced ROS generation. SH-SY5Y cells were pretreated for 24 hours with DMSO or 50 *μ*M D3T. Cells were treated with or without the NADPH oxidase inhibitor DPI (20 nM) or the PKC inhibitor rottlerin (2 *μ*M) for 30 min and then exposed to 4 mg/mL BSA or AGE-BSA for 16 hours. ROS generation is determined through DCF fluorescence. Results are from three independent experiments (*n* = 9). Bar values are expressed as mean ± SD. Letters that are not the same are significantly different (*P* < 0.05).

 AGE-BSA caused a significant (*P* > 0.0001 versus BSA control) increase in ROS, an effect abrogated by the NADPH oxidase-inhibitor DPI and the PKC inhibitor rottlerin (Figure 2). We tested if D3T potentiates AGE-induced ROS generation. Interestingly, AGE-induced ROS levels were higher after D3T pretreatment than DMSO pretreatment (*P* > 0.0001). This effect was also completely suppressed by DPI and rottlerin. Taken together, these data suggest that the potentiation of AGE-induced ROS by D3T is specifically through the PKC-NADPH oxidase pathway.

## 4. Discussion

Neuronal ROS generation in diabetes is increased by AGEs [38], which induce superoxide radical production through a pathway that connects RAGE and NADPH oxidase [39, 40]. Suppression of oxidative damage in diabetic neuropathy models may provide insight into possible treatments for this complication. Antioxidants like the green tea flavonoid EGCG [41] as well as *α*-lipoic acid, 17*β*-estradiol, and NAC [27] protect neuroblastoma SH-SY5Y cells against AGE-BSA. We previously reported that NAC increases intracellular GSH approximately threefold and suppresses the effects of AGEs on cell viability, neurite degeneration, and oxidative damage [36], suggesting that the NAC-mediated mechanism of protection is potent and deserving of further study. Because NAC increases GSH by providing cysteine, the limiting amino acid for GSH synthesis, we hypothesized that finding alternative routes to increase intracellular GSH may highlight other mechanisms for cytoprotection against AGEs.

 We tested if chemical induction of the endogenous antioxidant system, which includes GSH, promotes SH-SY5Y cell resistance to oxidative damage. We chose the compound D3T as it potently upregulates GSH levels while causing no independent negative effects in SH-SY5Y cells in culture. We found that D3T promoted cellular protection against H_2_O_2_. Because AGE-induced oxidative damage is mediated by H_2_O_2_ [27] and suppressed by GSH augmentation [36], we hypothesized that D3T protects SH-SY5Y cells against AGE-BSA. Surprisingly, D3T potentiated the cytotoxic and GSH-depleting effects of AGE-BSA. D3T had no influence on neurite degeneration or protein carbonylation; further consideration pointed to the possibility that worsening of these outcomes may not be feasible in this model. Our studies have not shown complete neurite degeneration, especially during a relatively short timeframe like that in this study. AGE-BSA exposure for 24 hours might have already caused neurite density to decline to its relative minimum, preventing D3T from causing further degeneration. Similarly, AGE-BSA caused a substantial increase in protein carbonylation, and this was not potentiated by D3T. AGE-induced protein oxidation is robust and effective, and conceivably, few opportunities for carbonylation might remain. In all, the antioxidant D3T is not protective against AGE-induced oxidative damage in SH-SY5Y cells.

 To test ROS generation in this model, we first confirmed previous findings by Nitti et al., showing that AGEs induce oxidative stress in a PKC and NADPH oxidase-dependent manner [19]. We further demonstrated that D3T potentiated AGE-induced ROS formation, which was suppressed by inhibition of PKC and NADPH oxidase. This suggests that D3T intensifies AGE-induced oxidative stress by specifically modulating the PKC-NADPH oxidase pathway. Nitti et al. found similar trends for RA, which increases p47^phox^ expression and PKC activity in SH-SY5Y cells [19] and increases cellular responsiveness to AGEs. We aim to perform future studies to test if this mechanism is similar to that of D3T.

In addition to D3T, our results showed that BSA also increased intracellular GSH. These data are supportive of the work by Cantin et al., demonstrating that albumin increases intracellular GSH, likely due to its endocytosis, degradation, and the utilization of its 34 cysteines [42]. Cells can internalize glycated albumin [43], suggesting that the GSH concentration differences between cells treated with BSA and AGE-BSA is not due exclusively to diminished biosynthesis. However, it is unclear if this process is affected by SH-SY5Y, so we will study this mechanism in our cells.

These findings are novel for a compound like D3T. In another study, the green tea polyphenol EGCG, which stimulates endogenous antioxidant defenses [44], was found to potentiate the cytotoxic effects of rotenone in SH-SY5Y cells [45]. However, EGCG treatment independently produced ROS in that study by Chung et al. This was further evidenced as the combination of EGCG and rotenone created a sum of ROS-producing mechanisms, resulting in higher toxicity. Interestingly, D3T induces ROS formation as part of its mechanism to increase antioxidant defenses [46, 47]. Although we did not observe a significant increase in DCF fluorescence by D3T alone, we cannot yet exclude the possibility that D3T subtly increases ROS in SH-SY5Y cells and causes sensitivity to AGEs in a more general manner.

 Chemical inducers of endogenous antioxidant defenses are considered a potential therapeutic target for many neurodegenerative diseases that have an oxidative stress component. This includes diabetic neuropathy, for which some studies show a protective role of these compounds. However, many studies have used high glucose as the primary stressor in models of diabetic complications. Here, we used AGEs and found that the compound D3T potentiates AGE-induced oxidative damage. In the context of these findings, it is imperative to study how D3T and similar compounds affect AGE-induced oxidative damage in additional cell and animal models before phytochemical antioxidants can be considered as useful in therapy for diabetic patients.
